# Ultrasensitive photoelectrochemical immunosensor based on floral cluster SnS_2_/ZnCdS heterostructure for the detection of CA199

**DOI:** 10.3389/fbioe.2025.1584456

**Published:** 2025-06-11

**Authors:** Hui Zhou, Qingqing Guo, Xin Zhang, Tingting Chu, Wen Zhang, Qing Liu, Linlin Cao

**Affiliations:** ^1^ Zibo Central Hospital Affiliated to Binzhou Medical University, Zibo, Shandong, China; ^2^ PKUCare Luzhong Hospital, Zibo, Shandong, China; ^3^ School of Chemistry and Chemical Engineering, Shandong University of Technology, Zibo, Shandong, China

**Keywords:** SnS2/ZnCdS, flower-like morphology, heterojunction, photoelectrochemical immunosensor, CA199

## Abstract

The early and accurate detection of tumor markers is crucial for cancer diagnosis, prognosis, and treatment monitoring. Carbohydrate antigen 199 (CA199), as a key biomarker of pancreatic, gastric, and colorectal cancers, is widely used in the clinical management. The development of sensitive, rapid and cost-effective detection methods for CA199 is of paramount importance in improving early detection rates and patient outcomes. In this study, we present a novel photoelectrochemical (PEC) immunosensor based on a SnS_2_/ZnCdS heterostructure designed for the ultrasensitive detection of CA199. The unique heterojunction between SnS_2_ and ZnCdS enhances photocurrent generation by effectively suppressing charge recombination and improving charge separation. Furthermore, the flower-like morphology of the heterostructure further boosts light absorption and photogenerated carrier transport, resulting in significantly enhanced sensor performance. This label-free PEC immunosensor exhibits outstanding stability, reproducibility and selectivity, with a broad detection range from 0.01 to 1000 U/mL and an ultra-low detection limit of 1.00 × 10^−3^ U/mL. These features demonstrate the potential of this sensor as a powerful tool for sensitive CA199 detection, offering promising applications in cancer diagnostics and monitoring.

## 1 Introduction

The early detection of tumor markers plays a pivotal role in the diagnosis, prognosis, and treatment of cancers. Among the various tumor markers, Carbohydrate Antigen 199 (CA199) has become a widely recognized biomarker, particularly for pancreatic, gastric, and colorectal cancers (Takada et al., 1993; Lee et al., 2020). Elevated levels of CA199 in serum are strongly associated with the presence and progression of these malignancies, making its detection critical for early diagnosis, recurrence monitoring, and treatment planning (Li Z. et al., 2022; Su et al., 2022; Zhou and Zhao, 2024). However, current detection methods for CA199 face numerous challenges, such as long detection times, high costs, and relatively low sensitivity. Therefore, there is a pressing need to develop more sensitive, rapid, and cost-effective CA199 detection techniques.

Traditional CA199 detection methods, including electrochemical immunoassays (Qi et al., 2024), the surface-enhanced Raman scattering (SERS)-based immunoassay (Li X. et al., 2022), electrochemiluminescent immunoassay (Gong et al., 2024), enzyme-linked immunosorbent assays (ELISA) (Fainberg et al., 2024), and radioimmunoassay (Kaur et al., 2017), have shown potential in clinical applications. However, these techniques are often limited by environmental contamination, high background signals, and expensive equipment. For instance, ELISA, while widely used, requires time-consuming procedures and complex reagents, making it unsuitable for point-of-care applications. SERS-based methods, although highly sensitive, rely heavily on specialized instrumentation and may suffer from low reproducibility due to the instability of the SERS substrate. Electrochemiluminescent immunoassays, though promising, typically require expensive chemical reagents and sophisticated setups. These limitations underscore the need for a more efficient detection platform with better sensitivity, simplicity, and low cost. Photoelectrochemical (PEC) biosensing technology has emerged as a promising alternative due to its inherent advantages, such as high sensitivity, low background interference, simple instrumentation and easy miniaturization (Zhao et al., 2015). PEC sensors utilize the interaction between biomolecules and the analyte to modulate the photocurrent generated by the light excitation of semiconductor materials. By employing light as the excitation source and measuring the resulting photocurrent, PEC biosensors effectively reduce background noise and enhance sensitivity, making them ideal for biomarker detection.

Recent advances in PEC biosensors have focused on the development of new photoactive materials to improve the performance of these sensors. Common materials such as TiO_2_, CdS, and ZnO have been widely investigated for PEC biosensing applications (Tereshchenko et al., 2016; Ming Qiao et al., 2020; Wang et al., 2024). However, many of these materials have limitations, such as poor light absorption, inefficient charge separation, and low photocurrent generation. For example, TiO_2_, while widely used, suffers from limited visible light absorption and inefficient charge separation (Akira Fujishima and Tryk, 2000). CdSe and CdS exhibit good optical properties but often face issues with low stability and high recombination rates of photogenerated carriers (Wei Li et al., 2022). ZnO is another commonly used material but is constrained by its wide bandgap and the challenges associated with improving photocurrent efficiency under visible light (Raha and Ahmaruzzaman, 2022). Among these, tin disulfide (SnS_2_) has attracted significant attention as a promising material for PEC biosensors due to its low cost, natural abundance, and stability under various conditions. However, SnS_2_ has a narrow bandgap (∼2.35 eV), which allows it to absorb visible light, but its weak photon absorption and low photocurrent generation significantly limit its application in PEC sensors (Lee, 2005).

Given the limitations of single material systems, the construction of heterojunctions has emerged as a powerful strategy to enhance the photocurrent response in PEC biosensors (Guo et al., 2023). A well designed heterostructure can effectively suppress charge recombination, improve charge separation, and enhance the photocatalytic activity of the materials. ZnCdS is also a narrow bandgap semiconductor with exceptional photocatalytic activity, high visible light absorption, and efficient electron transport properties. Therefore, we select Zinc cadmium sulfide (ZnCdS), an N-type semiconductor with a tunable bandgap, high photocatalytic activity, and exceptional visible light absorption to construct heterojunction with SnS_2_. The combination of SnS_2_ with ZnCdS in a heterojunction structure provides a highly synergistic effect, where the band structures of SnS_2_ and ZnCdS are highly compatible, leading to efficient electron transfer and reduce the recombination of photogenerated carriers, thereby enhancing photocurrent generation. Furthermore, the unique flower-like morphology of this heterojunction improves light absorption and facilitates the transport of photogenerated carriers, resulting in enhanced photocurrent generation.

Building on these principles, this study presents a novel label-free PEC immunosensor platform for the ultrasensitive detection of the tumor marker CA199, utilizing SnS_2_/ZnCdS as photosensitive substrates. The heterojunction design and the flower cluster morphology not only optimize light absorption and carrier transport but also effectively minimize electron-hole pair recombination, resulting in a substantial increase in photocurrent intensity compared to individual components. The resulting PEC sensor demonstrates remarkable stability, reproducibility, and selectivity, offering a promising approach for the practical and sensitive detection of tumor markers like CA199. Furthermore, this work highlights the potential of PEC sensors as a viable and highly efficient alternative to traditional diagnostic methods, providing a new pathway for cancer diagnostics and monitoring in clinical settings.

## 2 Materials and methods

### 2.1 Materials

The carbohydrate antigen (CA199), CA199 antibody (anti-CA199), carcinoembryonic antigen (CEA), and neuron specific enolase (NSE) used in this work were purchased from Shanghai Lingchao Biotechnolog Biotechnology y Co., LTD. Cardiac troponin I (CTnI) was purchased from Dingguo Changsheng Co., LTD. Indium tin oxide (ITO) (resistivity 10ω/sq) glass is purchased from China Nanhua Xiangcheng Technology Co., LTD. Tin chloride pentahydrate (SnCl_4_·5H_2_O), zinc nitrate hexahydrate (Zn(NO_3_)_2_·6H_2_O) and cadmium nitrate tetrahydrate (Cd(NO_3_)_3_·4H_2_O) were purchased from Shanghai Maclin Biochemical Technology Co., LTD. Thiourea (CH_4_N_2_S) from Tianjin Zhiyuan Chemical Reagent Co., LTD. 1-(3-dimethylaminopropyl) -3-ethylcarbodiimide hydrochloride (EDC) and N-hydroxysuccinimide (NHS) are from Aldeen Reagent (Shanghai) Co., LTD. Ascorbic acid (AA) and bovine serum albumin (BSA, 96%–99%) were purchased from Beijing Sigma-Aldrich Reagent Co., LTD. Anhydrous ethanol (C_2_H_5_OH) was purchased from Sinopod Group. Phosphate buffer solutions (PBS, 0.1 mol/L KH_2_PO_4_ and 0.1 mol/L Na_2_HPO_4_) were used for photoelectrochemical measurements. A solution prepared with 0.10 mol/L KCl and 5.0 mmol/L [Fe(CN)_6_]^3-/4-^ was used as the electrolyte for electrochemical impedance spectroscopy (EIS). Ultrapure water resistivity (≥18.2 MΩ) is obtained from the Millipore water purification system. All chemical reagents are analytical grade and can be used directly without further purification.

### 2.2 Equipment

The photocurrent signal was tested using the electrochemical workstation zenium-ecw. Scanning electron microscope (SEM) images were obtained by Field emission Scanning electron Microscope (Quanta, FEI). Electrochemical impedance spectroscopy (EIS) analysis was performed on CHI660E electrochemical workstation of Shanghai Chenhua Instrument Co., LTD. X-ray powder diffraction (XRD) patterns were obtained by D8 Advance X-ray diffractometer (Bruker AXS, Germany). High-resolution transmission electron microscopy (HRTEM) images were taken by Tecnai G2 F20S-TWIN Transmission Electron Microscopy (Field electron and Ion Co., United States of America). Analysis of the scale FS/JA (Shanghai Youke), magnetic stirrer RG-18 (Gongyi Yuhua), drying box DGG-9070B (Shanghai Senxin), Muffle furnace KSL-1200X (Hefei Kejing), vacuum drying box DZG-6020 (Shanghai Senxin), centrifuge H1850 (Xiangtan Xiangyi), high temperature sintering furnace NBD-O series (He Nanobadi) for material preparation.

### 2.3 Synthesis of SnS_2_ and ZnCdS

SnS_2_ nanosheets were prepared by hydrothermal method. Firstly, SnCl_4_·5H_2_O (0.35 g) was dispersed in 30 mL deionized water. A transparent solution was formed under magnetic stirring and 0.40 g CH_4_N_2_S was added as sulfur source. The resulting mixture was then transferred to a 50 mL autoclave and reacted at 180°C for 24 h; The product was cleaned with secondary deionized water and ethanol three times each. Finally, SnS_2_ was dried in 60°C oven for 24 h to obtain yellow powder.

Add 0.379 g Zn(NO_3_)_2_·6H_2_O, 0.617 g Cd(NO_3_)_3_·4H_2_O, 0.984 g CH_4_N_2_S and 20 mL ultra-pure water into 100 mL beaker and stir ultrasonic until completely dissolved. The mixed solution was transferred to a polytetrafluoroethylene autoclave, reacted at 160°C for 5 h, centrifuged with ethanol and deionized water, washed three times each, and dried in an oven at 120°C for 12 h to obtain orange powdered ZnCdS.

### 2.4 Construction of the PEC immunosensor

**FIGURE 1:**
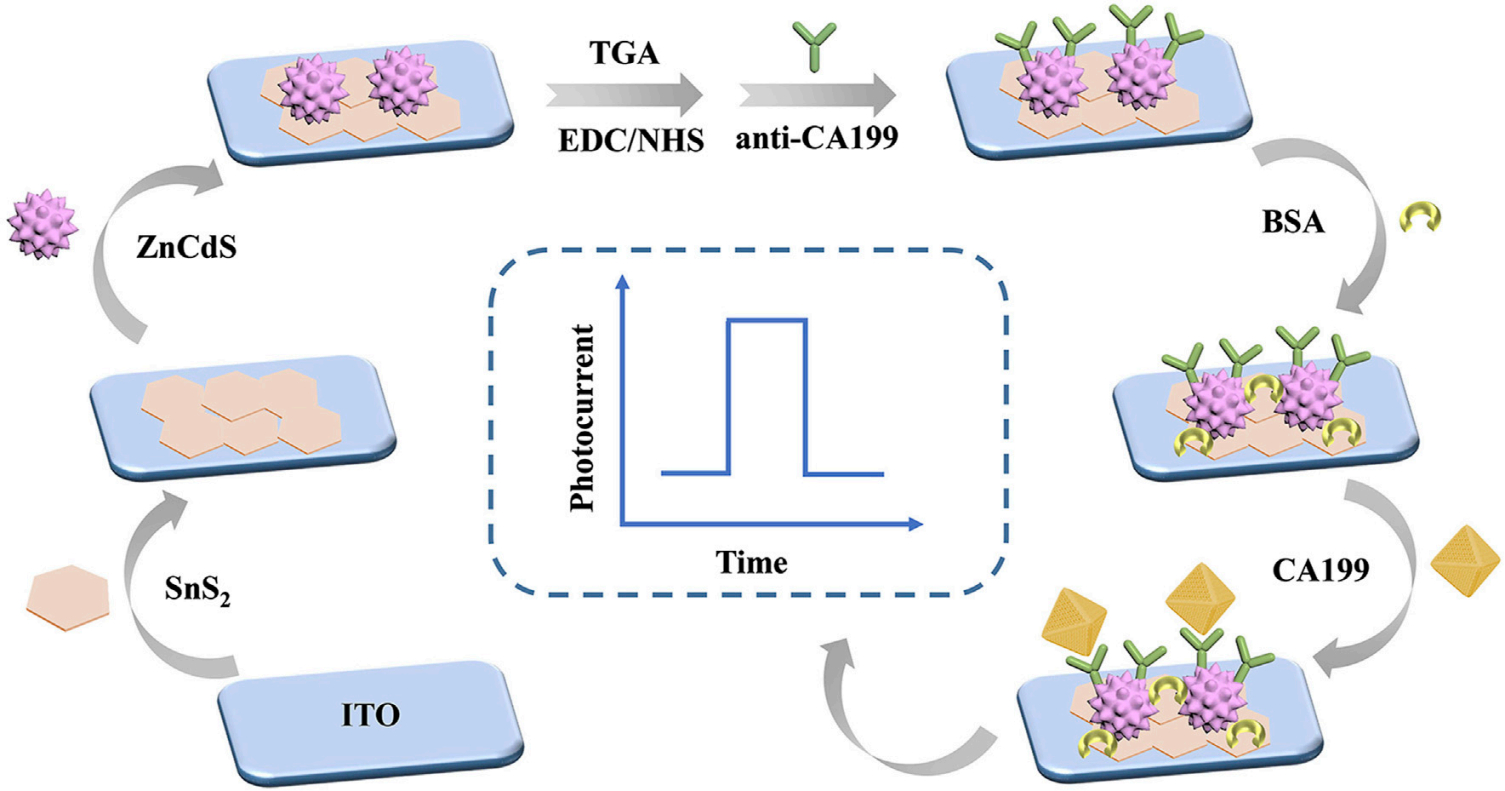
Construction process of PEC immunosensor for CA199 detection.

As shown in Figure 1, indium tin oxide (ITO)-coated glass slides (2.0 × 0.8 cm^2^) served as the substrate and working electrode for all photoelectrochemical measurements. Prior to use, the ITO substrates were sequentially cleaned in acetone, ethanol, and deionized water (30 min each) followed by oven drying. The upper surface of ITO electrode was first modified with 8 μL SnS_2_ (8 mg/mL) and dried in air to prepare the working electrode. The electrode surface was added with 8 μL ZnCdS (8 mg/mL) and dried in air. The SnS_2_/ZnCdS heterojunction ITO electrode was calcined in the Muffle furnace at 300°C for 30 min to ensure tight bonding of the materials. Thioglycollic acid (TGA) is added to provide carboxyl group. The carboxyl group of TGA was activated by adding 1- (3-dimethylaminopropyl) -3-ethylcarbodiimide hydrochloride (EDC)/n-hydroxysuccinimide (NHS) to the electrode; Then, 6 μL anti-CA199 (1 U/mL) was modified on the electrode. To block the non-specific active site, 3 μL of 1 wt% bovine serum albumin (BSA) was added to the electrode surface. Finally, the sensor platform was modified with different concentrations of CA199 to measure the photocurrent signal. To compare the photocurrent responses of SnS_2_, ZnCdS, and SnS_2_/ZnCdS heterostructures, photoelectrochemical sensors based on ITO/SnS_2_, ITO/ZnCdS, and ITO/SnS_2_/ZnCdS were fabricated, respectively.

### 2.5 PEC measurement

The photoelectric signal was measured using a photochemical workstation with a traditional three-electrode system. The electrodes were placed in a 0.1 mol/L phosphate buffered saline solution (PBS, pH = 7.38) containing ascorbic acid to collect the photocurrent signal, and a current-time curve was drawn. LED light source (Model: LSW-2) with an irradiance of 1200 W/m^2^ was employed as the irradiation source. During the PEC test, the light source lamp was turned on and off every 20 s, resulting in the opening and closing of the photoelectric signal at 0 V voltage.

## 3 Results and discussion

### 3.1 Synthesis and characterization

**FIGURE 2:**
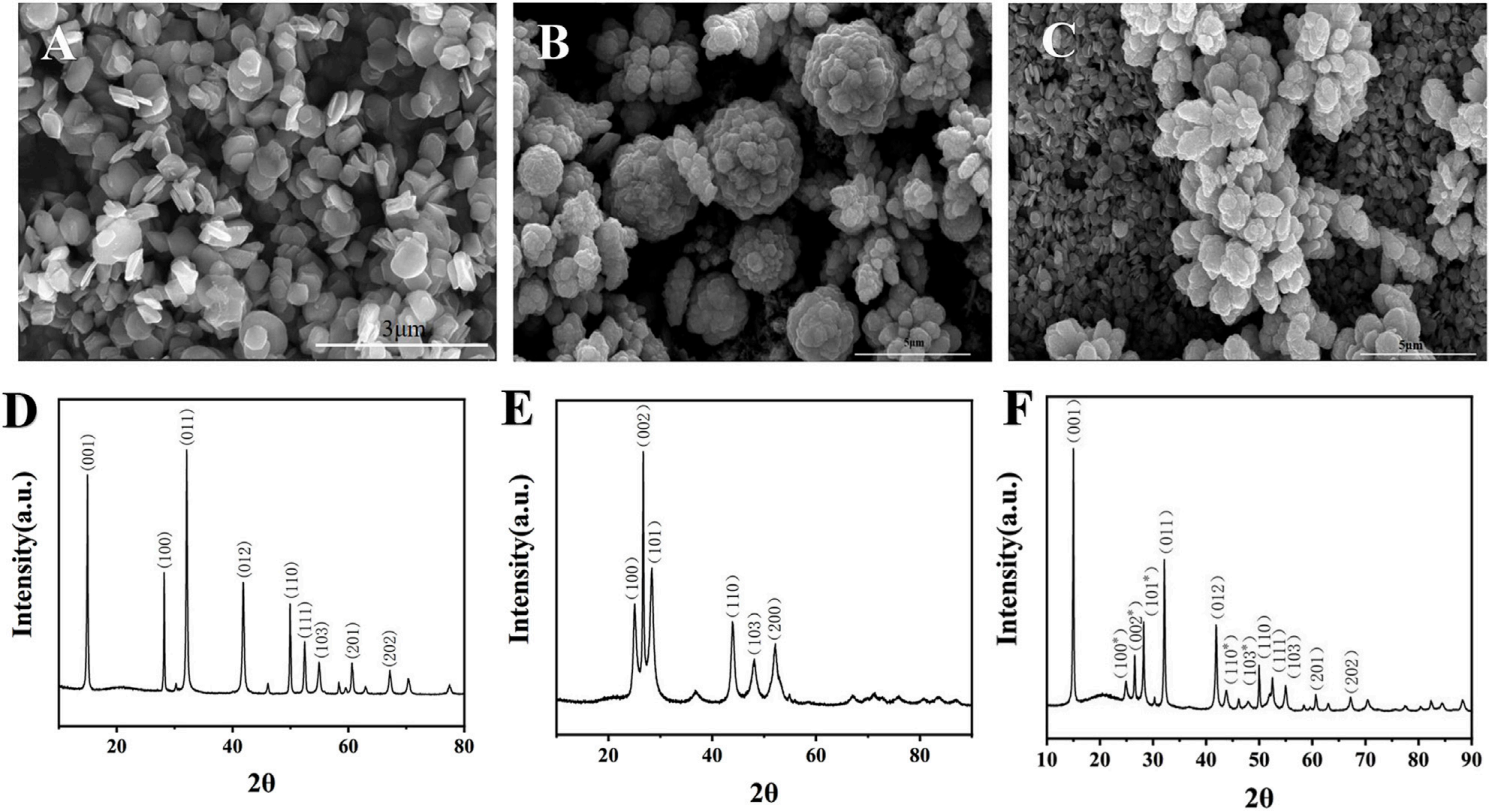
TEM and XRD characterization of SnS_2_, ZnCdS and SnS_2_/ZnCdS. **(A)** SEM image of SnS_2_; **(B)** SEM images of ZnCdS; **(C)** SEM images of SnS_2_/ZnCdS; **(D)** XRD spectra of SnS_2_; **(E)** XRD spectra of ZnCdS; **(F)** XRD spectra of SnS_2_/ZnCdS.

The materials were characterized by scanning electron microscopy (SEM). As shown in Figure 2A, the prepared SnS_2_ nanosheets were hexagonal in shape, the size was basically the same, and the diameter was about 500 nm. Due to their larger surface area, SnS_2_ nanosheets not only provide more binding sites, but also facilitate light capture and electron transfer (Zhou et al., 2020). ZnCdS have an average diameter of about 4 μm and appear as clusters of flowers (Figure 2B). The larger surface area of ZnCdS provides a richer contact interface for photoactive reactions, promotes light absorption, and provides more space and active interface sites. Supplementary Figure S1 presents the Ultraviolet-Visible diffuse reflectance spectroscopy (UV-Vis-DRS) of ZnCdS, demonstrating strong absorption centered at 450 nm with a broad spectral response extending across the visible range. Figure 2C shows that flower-cluster ZnCdS are tightly bound to SnS_2_ nanosheets.

X-ray diffraction (XRD) was used to determine the crystal structure of the sample (Pandey et al., 2021). As shown in Figure 2D, SnS_2_ showed relatively strong diffraction peaks at positions such as 15.03, 28.19, 32.21, 41.99, 50.11, 52.62, 55.14, 60.82 and 67.38, corresponding to SnS_2_ crystal (001), (100), (011), (012), (110), (111), (103), (201) and (202), and no impurity diffraction peak, indicating that the prepared SnS_2_ has a high purity. The characteristic diffraction peaks of ZnCdS appeared at 24.83, 26.53, 28.2, 43.73 and 51.87, corresponding to the (100), (002), (101), (110), (103) and (112) planes, meaning that the ZnCdS has been successfully synthesized (Figure 2E). The diffraction peaks of the SnS_2_/ZnCdS samples exhibited a strong correspondence with a single component, indicating the successful formation of SnS_2_/ZnCdS composites, as depicted in Figure 2F.

**FIGURE 3:**
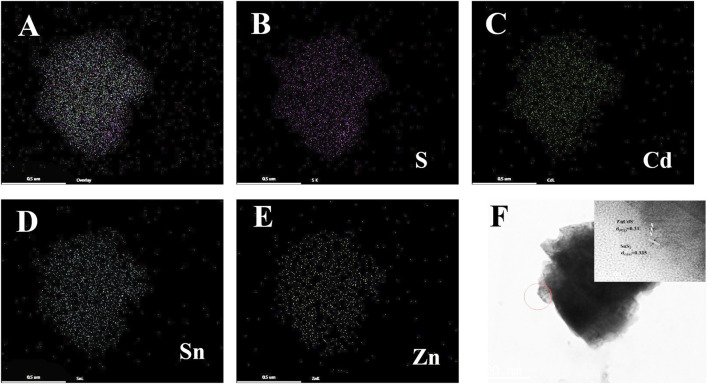
SnS_2_/ZnCdS element mapping and HRTEM mapping. **(A)** Elemental mapping of SnS_2_/ZnCdS; **(B–E)** mapping images of S, Cd, Sn and Zn; **(F)** HRTEM images of SnS_2_/ZnCdS.

The element mapping image of SnS_2_/ZnCdS composite was shown in Figures 3A–E, and the elements S, Cd, Sn and Zn were evenly distributed in the composite. High-resolution transmission electron microscopy (HRTEM) is crucial for the characterization of materials. HRTEM of SnS_2_/ZnCdS was observed in Figure 3F. The different lattice configurations of 0.335 nm and 0.31 nm correspond to the (110) crystal plane of SnS_2_ and the (022) crystal plane of ZnCdS (Li Y. et al., 2022) respectively, confirming that the heterojunction composites have been successfully prepared.

### 3.2 Characterization of unlabeled CA199 PEC immunosensors

The photoelectric conversion performance of a heterojunction is critically dependent on its band alignment configuration. The energy band structure of the material was systematically characterized using Ultraviolet-Visible Diffuse reflectance spectroscopy (UV-Vis-DRS) combined with Mott-Schottky measurements to determine the conduction band edge positions. The band gap (E_g_) was calculated using the fundamental relation, E_g_ = E_VB_ - E_CB_, where E_VB_ and E_CB_ represent the valence band and conduction band energies, respectively. As shown in Supplementary Figure S2, the SnS_2_ exhibited a conduction band position at −1.04 eV, a valence band position at 1.44 eV, and a bandgap of 2.48 eV. Similarly, Mott-Schottky analysis revealed that ZnCdS possessed a conduction band position at −1.14 eV, a valence band position at 0.93 eV, and a bandgap of 2.07 eV. These precisely determined band parameters provide essential guidance for the rational design and performance optimization of heterojunction systems, particularly regarding their charge separation and transfer capabilities.

**FIGURE 4:**
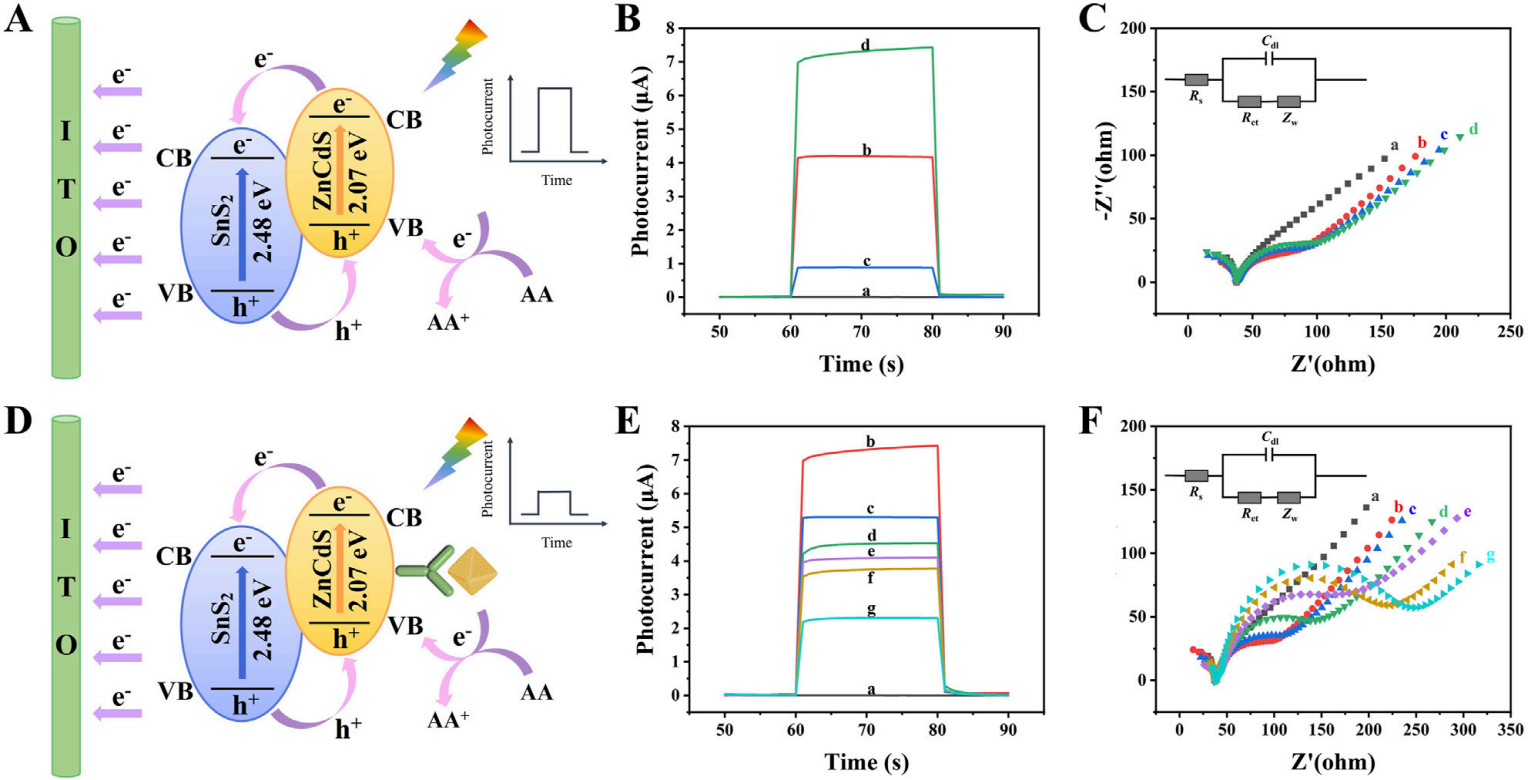
Photoelectric chemical characterization of PEC immunosensor. **(A)** The electron transfer mechanism in PEC immunosensors without antigens/antibodies; **(B)** The photocurrent response curve of the photoactive material and **(C)** EIS Nyquist plots of (a) ITO, (b) ITO/SnS_2_/ZnCdS,(c) ITO/SnS_2_, and (d) ITO/ZnCdS. **(D)** The electron transfer mechanism in PEC immunosensors with antigen-antibody complexes; **(E)** Photocurrent response curve and **(F)** EIS Nyquist plots of (a) ITO, (b) ITO/SnS_2_/ZnCdS, (c) ITO/SnS_2_/ZnCdS/TGA, (d) ITO/SnS_2_/ZnCdS/TGA/EDC/NHS, (e) ITO/SnS_2_/ZnCdS/TGA/EDC/NHS/anti-CA199, (f) ITO/SnS_2_/ZnCdS/TGA/EDC/NHS/anti-CA199/BSA, (g) ITO/SnS_2_/ZnCdS/TGA/EDC/NHS/anti-CA199/BSA/CA199.

The photogenerated carrier transfer mechanism of the PEC immunosensor constructed in this study is illustrated in Figure 4A. The band structures of SnS_2_ and ZnCdS exhibit a staggered alignment in terms of their bandgap widths, conduction band positions, and valence band positions, forming a type-II heterojunction. According to the semiconductor energy band theory, under the excitation of light, the carrier absorption energy in the valence band of SnS_2_ and ZnCdS transitions to the conduction band. Due to the lower Fermi energy level of SnS_2_ compared to ZnCdS, the Fermi energy level difference is generated at the heterojunction interface, driving the photogenerated carriers to migrate from the conduction band of ZnCdS to that of SnS_2_. In addition, the oxidation reaction of ascorbic acid (AA), which is an electron donor in the electrolyte solution, replenishes holes in the valence band and reduces the recombination of e^−^/h^+^ by preventing electron backflow from the conduction band (Xiaoquan et al., 2007; Mei et al., 2024). Therefore, the SnS_2_/ZnCdS heterojunction not only accelerates the separation and migration of photogenerated carriers but also significantly enhances the photoelectric conversion efficiency.

The photocurrent time curve and EIS curve were often used to characterize the layer modification process of PEC sensors. The experimental data presented in Figures 4B,C, and Supplementary Table S1 revealed that the bare ITO produced essentially no detectable signal (curve a). When SnS_2_ was modified on the electrode, the photoelectric signal was significantly enhanced (curve c), which proved that SnS_2_, as an excellent N-type semiconductor photosensitive material, had good photoelectric response under visible light. When only ZnCdS were modified on the electrode, the photocurrent signal was weak (curve d). When SnS_2_/ZnCdS were modified on the electrode, a relatively good photocurrent response was obtained (curve b).

When the non-conductive substances such as BSA and CA199 were gradually modified on the electrode, the photocurrent decreases successively due to the obvious steric hindrance of the immune substances on electron transfer as shown in Figures 4D–F, confirming the successful construction of the PEC sensor. Electrochemical impedance spectroscopy (EIS) was an important method to prove the successful preparation of PEC immunosensors. The diagram in Figure 4F represents the fitted equivalent circuit model, where *C*_dl_ is the internal resistance of the electrolyte between the reference electrode and the working electrode, *R*_s_ is the double layer capacitance, *R*_et_ is the charge transfer resistance, and *Z*_w_ is the Warburg impedance. During the measurements, 5.0 mmol/L [Fe(CN)_6_] ^3-/4-^ (0.10 mol/L KCl) was used as the electrolyte solution. The semicircle represents the electron transfer resistance (*R*_et_), which was an important indicator in the sensor manufacturing process and reflects the interface characteristics of different modified electrodes (Randviir and Banks, 2022). As shown in Figure 4F and Supplementary Table S2, the SnS_2_/ZnCdS heterojunction had a small electron transfer resistance, which indicated that the heterojunction had a significant e^−^/h^+^ separation capability (curve b). When anti-CA199, BSA, and CA199 were sequentially modified, *R*_et_ increased, indicating that the non-conductive protein was successfully attached to the electrode. The change trend of EIS Nyquist was consistent with the time-photocurrent response curve, indicating that the immune sensor was successfully constructed.

### 3.3 Optimization of experimental conditions

In order to improve the sensor performance, SnS_2_ concentration, ZnCdS concentration, ascorbic acid (AA) concentration and PBS electrolyte pH value all affect the current signal size during the construction of the photoelectric chemical sensor. Therefore, it is necessary to optimize the experimental conditions to obtain the best photocurrent signal.

**FIGURE 5:**
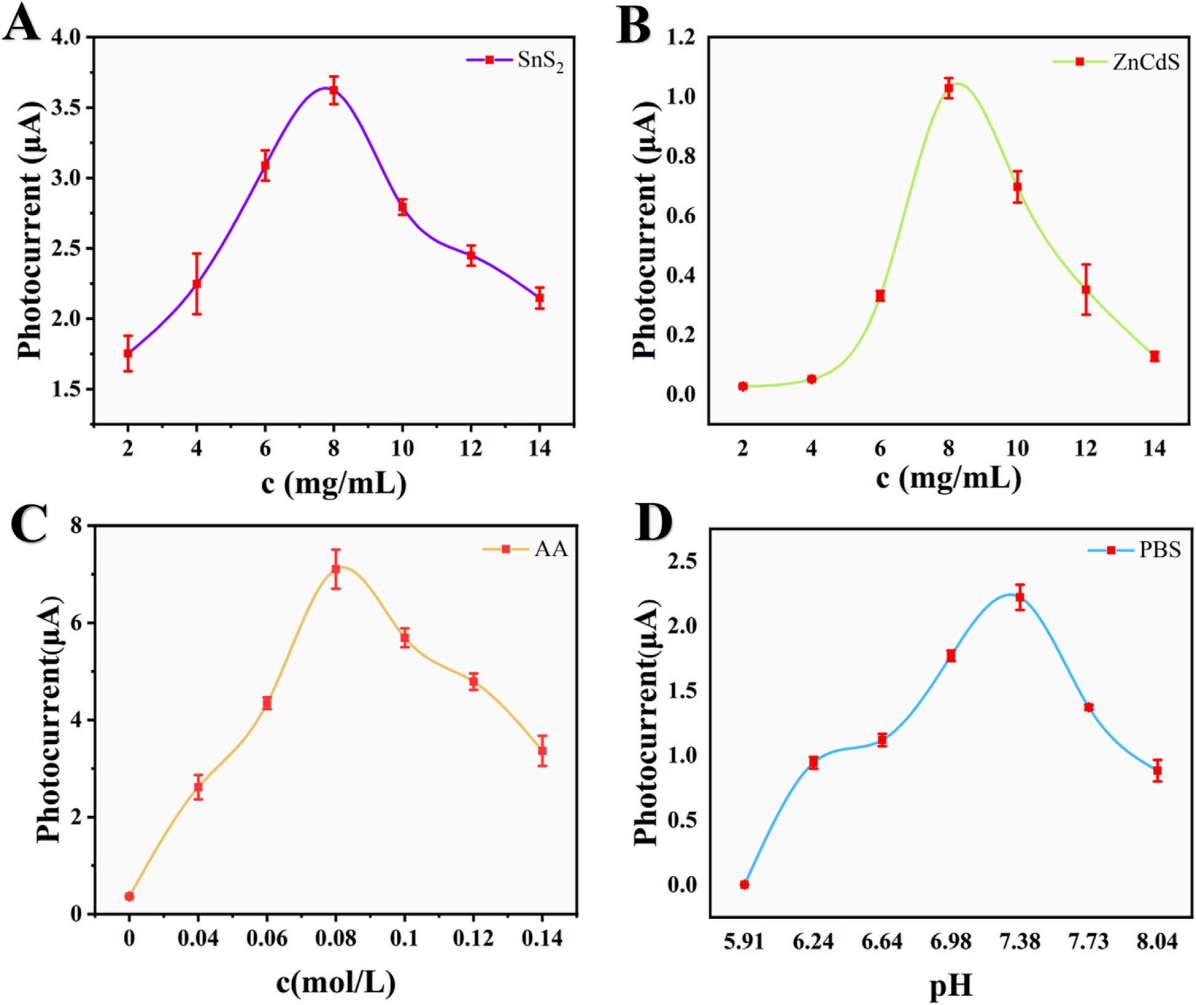
Optimization of experimental conditions. **(A)** Concentration of SnS_2_, **(B)** Concentration of ZnCdS, **(C)** Concentration of AA, **(D)** PH of PBS. All data reported are presented as the mean ± SD (n = 3).

The concentration of the substrate material affects the photoelectric current signal. Three sets of substrate materials with different concentrations were modified on the glassy carbon electrode surface to construct a photoelectrochemical immunoassay sensor. The testing range of SnS_2_ concentration was 2–14 mg/mL, as shown in Figure 5A. As the concentration of SnS_2_ increased, the photoelectric current signal gradually increased, reaching a peak at 8 mg/mL (3.62 μA), followed by a rapid decline in signal strength. The optimal concentration of ZnCdS was also tested within the range of 2–14 mg/mL, as shown in Figure 5B. The optimal concentration of ZnCdS was 8 mg/mL, with the highest photoelectric current signal of 1.03 μA. AA was used as an electron donor for the photoelectrochemical sensor, and the photoelectric current signal changed accordingly with the concentration of AA. When the average concentration of AA was 0.08 mol/L, the photoelectric current signal reached its peak value of 7.10 μA (Figure 5C). As shown in Figure 5D, the photocurrent signal reaches its peak at pH 7.38, demonstrating that either acidic or alkaline conditions would impair protein activity and cause deactivation, whereas a neutral environment better maintains the functionality of antibody-antigen interactions. In summary, this study optimized the experimental conditions, including the concentration of SnS_2_, ZnCdS, AA, and the pH value of PBS, and finally determined the optimal experimental conditions of 8 mg/mL SnS_2_, 8 mg/mL ZnCdS, 0.08 mol/L AA, and pH 7.38.

### 3.4 PEC detection of CA199

**FIGURE 6:**
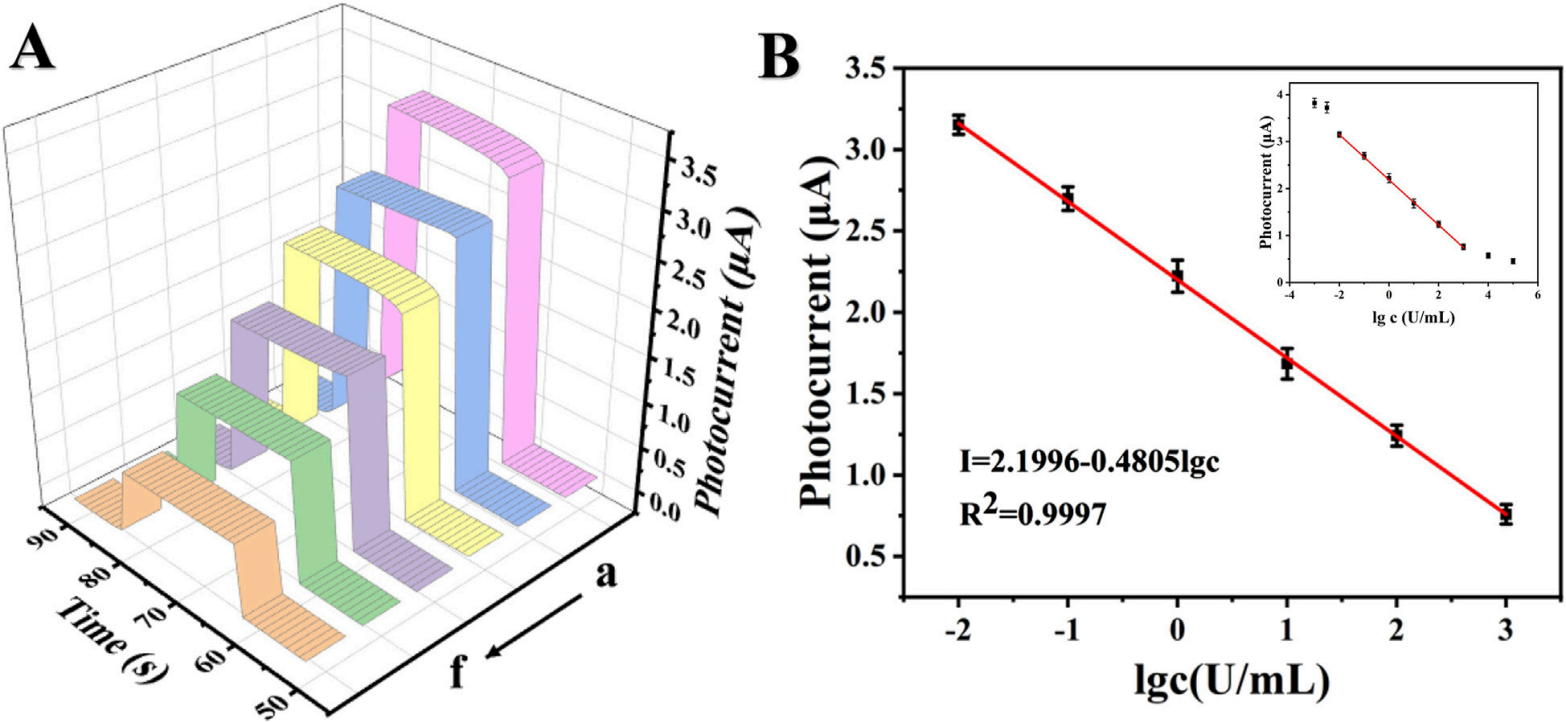
Photocurrent response curves and standard curves of CA199. **(A)** Photocurrent response curves of CA199 (from a to f: 0.01 U/mL, 0.1 U/mL, 1 U/mL, 10 U/mL, 100 U/mL and 1000 U/mL), **(B)** Standard curves of PEC immunoassay.

The detection of the tumor marker CA199 was based on a specific immune response between an antigen and an antibody. As depicted in Figure 6A, the photocurrent response of the PEC sensor exhibited a direct correlation with the concentration of CA199. Various concentrations of CA199 ranging from 0.01 U/mL to 1000 U/mL were detected under optimal experimental conditions. Upon data fitting, it was observed that the photocurrent signal demonstrates a clear linear relationship with the logarithm of CA199 concentration (Figure 6B). The regression equation for the standard curve was determined as: I (μA) = 2.1996–0.4805 lg c, yielding a high correlation coefficient value of 0.9997. By employing calculation methods, it has been determined that the limit of detection (LOD) for the PEC immune sensor was at an impressive level of 1.00 × 10^−3^ U/mL (S/N = 3). The PEC immunosensor prepared in this work has a wider detection range and lower detection limit compared to the CA199 reported in the literature (Table 1).

**TABLE 1:** Comparison of different detection methods for CA199.

Analysis methods	Linear range (U/mL)	Detection limit (U/mL)	Ref.
EC	1 × 10^−4^–70	3.30 × 10^−5^	Tan et al. (2022)
ECL	0.0005–200	1.30 × 10^−4^	Bahari et al. (2022)
SERS	5 × 10^−4^–1 × 10^2^	3.43 × 10^−4^	Jiang et al. (2021)
SPCE	0.01–40	0.07	Ibáñez-Redín et al. (2020)
Colorimetric Assay	8.6 × 10^−5^–1.4 × 10^−2^	3.50 × 10^−5^	Li et al. (2019)
EC	1 × 10^−5^–10	2.429 × 10^−6^	Qiu et al. (2022)
THz immunosensor	0.01–150	0.01	Lin et al. (2022)
Fluorescence	2.76 × 10^−2^–5.23 × 10^2^	1.58 × 10^−3^	Piloto et al. (2022)
PEC	0.01–1,000	1.00 × 10^−3^	This work

### 3.5 Stability, reproducibility and selectivity analysis of PEC immunosensors

**FIGURE 7:**
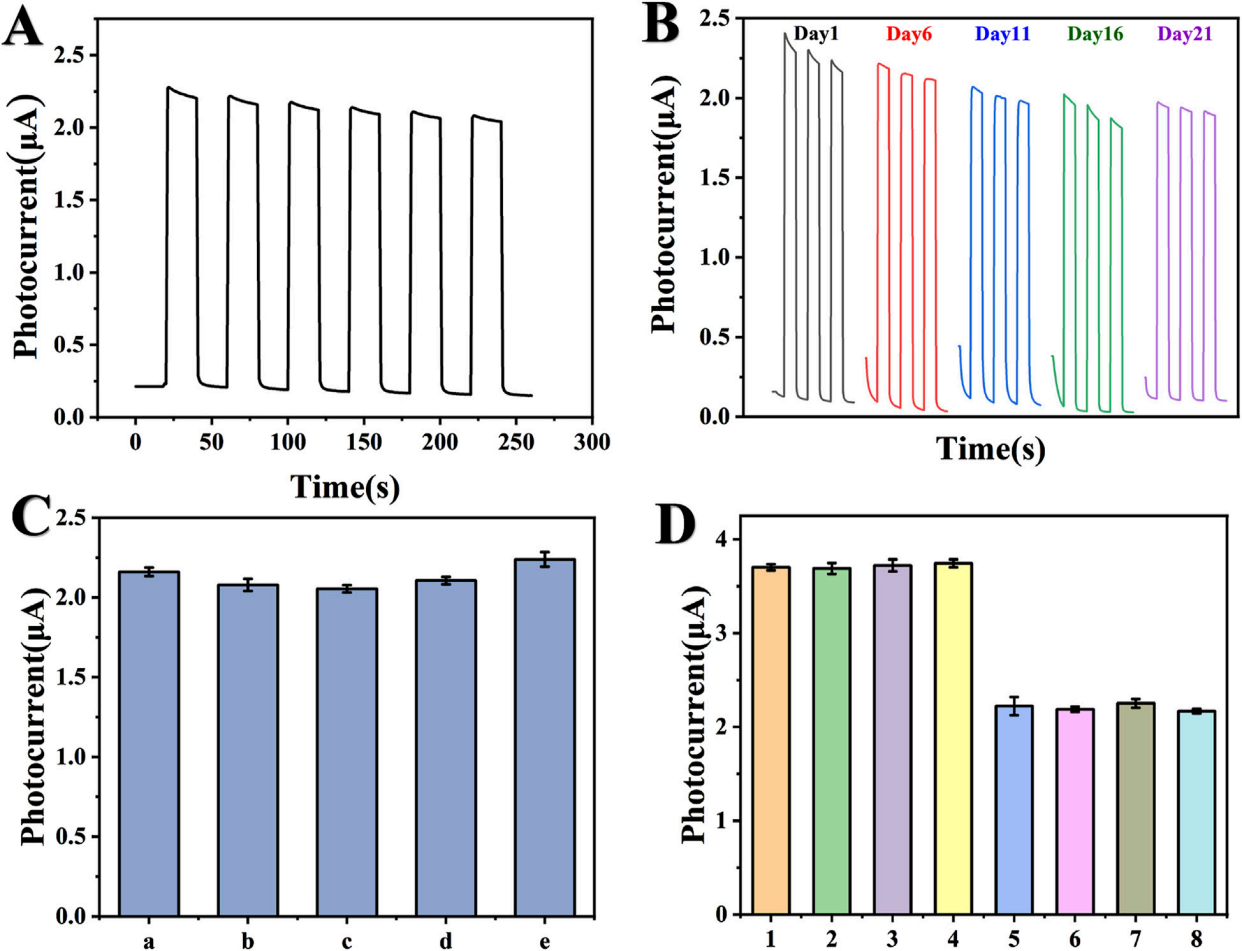
Stability, reproducibility, and selectivity of PEC immunosensor. **(A)** Stability assessment of the PEC immunosensor under on/off irradiation cycles (CA199 concentration 1 U/mL). The applied potential is 0 V), **(B)** Stability assessment of PEC immunosensors at different time periods, **(C)** Repeatability analysis of five groups of sensors constructed simultaneously, **(D)** Selective analysis of CA199 PEC immunosensors. The distractors added by each sensor are as follows: (1) blank samples, (2) 100 ng/mL CEA, (3) 100 ng/mL NSE, (4) 100 ng/mL CTnI, (5) 1 U/mL CA199, (6) 1 U/mL CA199 + 100 ng/mL CEA, (7) 1 U/mL CA199 + 100 ng/mL NSE, (8) 1 U/mL CA199 + 100 ng/mL CTnI.

The stability of the proposed PEC immunosensor was assessed through analysis of the photocurrent response curve. As depicted in Figure 7A, no significant variation in photocurrent response was observed over a period of 250 s, indicating excellent short-term stability with virtually unchanged chemical and optical properties of the sensor. To evaluate its long-term stability, the immunosensor was stored at 4°C, and electrical signal values were monitored every 5 days, with five consecutive measurements taken each time. As shown in Figure 7B, signal response value gradually decreased but stabilized at 85.59%. Experimental data demonstrate that the prepared immune sensor exhibits good stability. Additionally, as illustrated in Figure 7C, constructed PEC immune sensors exhibit excellent repeatability, electrodes a-e refer to sensors prepared under identical experimental conditions using CA199 as target detection object at concentration level of 0.1 U/mL. Low relative standard deviation (3.47%) confirms stable construction for this sensor analysis strategy while testing several typical interfering substances including carcinoembryonic antigen (CEA), neuron-specific enolase (NSE), and troponin I (cTnI). Figure 7D shows that the interferences (CEA, NSE, and cTnI) and the target test object CA199 coexist with each other with little interference, indicating that the immunoassay system has satisfactory selectivity.

### 3.6 Real sample analysis

In this study, we further employed the additive recovery experiment to assess the practical application potential of PEC sensors. Table 2 presents the results of three groups of human serum tests. The maximum relative standard deviation (RSD) was calculated as 2.33%, and the recovery rate ranged from 90% to 101%, indicating that the immunosensor exhibits promising utility for CA199 detection in authentic samples.

**TABLE 2:** Detection of CA199 in serum samples.

Sample	CA199(U/mL)	Addition (U/mL)	Result (U/mL)	RSD (%, n = 5)	Average recovery rate (%)
1	11.6	1	12.7, 12.2, 12.3, 12.4, 12.9	2.33	90
2	11.6	5	16.4, 15.9, 16.7, 16.8, 16.5	2.13	97.2
3	11.6	10	21.8, 21.5, 22.3, 21.7, 21.2	1.87	101

## 4 Conclusion

A label-free photochemical immunosensor for the detection of CA199 was developed using a specialized heterostructure composed of SnS_2_/ZnCdS. The unique floral cluster structure of ZnCdS enhances visible light absorption and provides more active sites for antibodies. The step-level transition of photogenerated electrons along SnS_2_/ZnCdS exhibits excellent electron transport performance. The constructed CA199 photoelectric chemical immunosensor in this study demonstrates a wide detection range (10^–2^ U/mL to 10^3^ U/mL) and a low detection limit (1.00 × 10^−3^ U/mL). Moreover, the PEC immunosensor shows good reproducibility, stability, and selectivity for the detection of tumor markers.

## Data Availability

The original contributions presented in the study are included in the article/Supplementary Material, further inquiries can be directed to the corresponding author.

## References

[B1] Akira Fujishima T. N. R. Tryk D. A. (2000). Titanium dioxide photocatalysis. J. Photochem. Photobiol. C Photochem. Rev. 1 (1), 1–21. 10.1016/s1389-5567(00)00002-2

[B2] Bahari D. Babamiri B. Moradi K. Salimi A. Hallaj R. (2022). Graphdiyne nanosheet as a novel sensing platform for self-enhanced electrochemiluminescence of MOF enriched ruthenium (II) in the presence of dual co-reactants for detection of tumor marker. Biosens. and Bioelectron. 195, 113657. 10.1016/j.bios.2021.113657 34607118

[B3] Fainberg H. P. Moodley Y. Triguero I. Corte T. J. Sand J. M. B. Leeming D. J. (2024). Cluster analysis of blood biomarkers to identify molecular patterns in pulmonary fibrosis: assessment of a multicentre, prospective, observational cohort with independent validation. Lancet. Respir. Med. 12 (9), 681–692. 10.1016/S2213-2600(24)00147-4 39025091

[B4] Gong Z. Lu B. Wang H. Ren X. Liu X. Ma H. (2024). Double-amplified electrochemiluminescence immunoassay sensor for highly sensitive detection of CA19-9 using a ternary semiconductor CdSSe. Anal. Chem. 96 (4), 1678–1685. 10.1021/acs.analchem.3c04690 38215346

[B5] Guo X. Zhang F. Zhang Y. Hu J. (2023). Review on the advancement of SnS2 in photocatalysis. J. Mater. Chem. A 11 (14), 7331–7343. 10.1039/d2ta09741a

[B6] Ibáñez-Redín G. Materon E. M. Furuta R. H. M. Wilson D. do Nascimento G. F. Melendez M. E. (2020). Screen-printed electrodes modified with carbon black and polyelectrolyte films for determination of cancer marker carbohydrate antigen 19-9. Microchim. Acta 187 (7), 417. 10.1007/s00604-020-04404-6 32613349

[B7] Jiang J. Liu H. Li X. Chen Y. Gu C. Wei G. (2021). Nonmetallic SERS-based immunosensor byintegrating MoS2 nanoflower and nanosheet towards the direct serum detection of carbohydrate antigen 19-9. Biosens. Bioelectron. 193, 113481. 10.1016/j.bios.2021.113481 34252705

[B8] Kaur S. Smith L. M. Patel A. Menning M. Watley D. C. Malik S. S. (2017). A combination of MUC5AC and CA19-9 improves the diagnosis of pancreatic cancer: a multicenter study. Am. J. Gastroenterology 112 (1), 172–183. 10.1038/ajg.2016.482 PMC5365072 27845339

[B9] Lee C. I. (2005). Optical properties of SnSz single crystals. Korean J. Mater. Res. 15 (3), 195–201.

[B10] Lee T. Teng T. Z. J. Shelat V. G. (2020). Carbohydrate antigen 19-9 - tumor marker: past, present, and future. World J. Gastrointest. Surg. 12 (12), 468–490. 10.4240/wjgs.v12.i12.468 33437400 PMC7769746

[B11] Li N.-S. Lin W.-L. Hsu Y.-P. Chen Y.-T. Shiue Y.-L. Yang H.-W. (2019). Combined detection of CA19–9 and MUC1 using a colorimetric immunosensor based on magnetic gold nanorods for ultrasensitive risk assessment of pancreatic cancer. ACS Appl. Bio Mater. 2 (11), 4847–4855. 10.1021/acsabm.9b00616 35021484

[B12] Li X. Ma J. Zhang Y. Xu L. Gu C. Wei G. (2022a). Reusable dual-functional SERS sensor based on gold nanoflowers-modified red phosphorus nanoplates for ultrasensitive immunoassay and degradation of CA19-9. Biosens. and Bioelectron. 207, 114148. 10.1016/j.bios.2022.114148 35286945

[B13] Li Y. Cao L. Shen C. Meng F.-N. Li Y. Wang S. (2022b). Heterostructure photoelectrochemical immunosensor based on flower-like refraction structure Cd-ZnIn2.2Sy sensitized 2D hexagonal SnS2 nanoplates for CA242 detection. Sensors Actuators B Chem. 367, 132186. 10.1016/j.snb.2022.132186

[B14] Li Z. Zhu H. Pang X. Mao Y. Yi X. Li C. (2022c). Preoperative serum CA19-9 should be routinely measured in the colorectal patients with preoperative normal serum CEA: a multicenter retrospective cohort study. BMC Cancer 22 (1), 962. 10.1186/s12885-022-10051-2 36076189 PMC9454113

[B15] Lin S. Wang Y. Peng Z. Chen Z. Hu F. (2022). Detection of cancer biomarkers CA125 and CA199 via terahertz metasurface immunosensor. Talanta 248, 123628. 10.1016/j.talanta.2022.123628 35660997

[B16] Mei L.-P. Xu J.-J. Tanjung A. P. Wang A.-J. Wu L. Song P. (2024). Integration of high-entropy nanozyme and hollow In2S3 nanotube heterostructures decorated with WO3 for ultrasensitive PEC aptasensing of highly toxic mycotoxin. Sensors Actuators B Chem. 414, 135952. 10.1016/j.snb.2024.135952

[B17] Ming Qiao J. Y. Qu L. Zhao B. Yin J. Cui T. Jiang L. (2020). Femtosecond laser induced phase transformation of TiO2 with exposed reactive facets for improved photoelectrochemistry performance. ACS Appl. Mater. and Interfaces 12 (37), 41250–41258. 10.1021/acsami.0c10026 32813491

[B18] Pandey A. Dalal S. Dutta S. Dixit A. (2021). Structural characterization of polycrystalline thin films by X-ray diffraction techniques. J. Mater. Sci. Mater. Electron. 32 (2), 1341–1368. 10.1007/s10854-020-04998-w

[B19] Piloto A. M. L. Ribeiro D. S. M. Rodrigues S. S. M. Santos J. L. M. Sampaio P. Sales M. G. F. (2022). Cellulose-based hydrogel on quantum dots with molecularly imprinted polymers for the detection of CA19-9 protein cancer biomarker. Mikrochim. Acta 189 (4), 134. 10.1007/s00604-022-05230-8 35247077

[B20] Qi F. Wu M. Liu S. Mu W. Wu C. Ren X. (2024). Ratiometric electrochemical immunosensor for the detection of CA199 based on the ratios of NiCo@Fc-MWCNTs-LDH and 3D-rGOF@Ag/Au complexes. Talanta 272, 125606. 10.1016/j.talanta.2023.125606 38394747

[B21] Qiu R. Dai J. Meng L. Gao H. Wu M. Qi F. (2022). A novel electrochemical immunosensor based on COF-LZU1 as precursor to form heteroatom-doped carbon nanosphere for CA19-9 detection. Appl. Biochem. Biotechnol. 194 (7), 3044–3065. 10.1007/s12010-022-03861-4 35334069

[B22] Raha S. Ahmaruzzaman M. (2022). ZnO nanostructured materials and their potential applications: progress, challenges and perspectives. Nanoscale Adv. 4 (8), 1868–1925. 10.1039/d1na00880c 36133407 PMC9419838

[B23] Randviir E. P. Banks C. E. (2022). A review of electrochemical impedance spectroscopy for bioanalytical sensors. Anal. Methods 14 (45), 4602–4624. 10.1039/d2ay00970f 36342043

[B24] Su J. Wang Y. Shao H. You X. Li S. (2022). Value of multi-detector computed tomography combined with serum tumor markers in diagnosis, preoperative, and prognostic evaluation of pancreatic cancer. World J. Surg. Oncol. 20 (1), 323. 10.1186/s12957-022-02785-x 36175918 PMC9520929

[B25] Takada A. Ohmori K. Yoneda T. Tsuyuoka K. Hasegawa A. Kiso M. (1993). Contribution of carbohydrate antigens sialyl Lewis A and sialyl Lewis X to adhesion of human cancer cells to vascular endothelium. Cancer Res. 53 (2), 354–361. 7678075

[B26] Tan Y.-Y. Sun H.-N. Liu M. Liu A. Li S.-S. (2022). Simple synthesis of PtRu nanoassemblies as signal amplifiers for electrochemical immunoassay of carbohydrate antigen 19-9. Bioelectrochemistry Amst. Neth. 148, 108263. 10.1016/j.bioelechem.2022.108263 36162334

[B27] Tereshchenko A. Bechelany M. Viter R. Khranovskyy V. Smyntyna V. Starodub N. (2016). Optical biosensors based on ZnO nanostructures: advantages and perspectives. A review. Sensors Actuators B Chem. 229, 664–677. 10.1016/j.snb.2016.01.099

[B28] Wang S. Liu Y. Chai Y. Yuan R. Liu H. (2024). An ultrasensitive photoelectrochemical biosensor based on AgBiS2/CdS photoanode and multiple signal amplification strategy for the detection of dibutyl phthalate plasticizer. Sensors Actuators B Chem. 414, 135945. 10.1016/j.snb.2024.135945

[B29] Wei Li X. C. Wang F. Dang Y. Liu X. Ma T. Li J. (2022). Pd single-atom decorated CdS nanocatalyst for highly efficient overall water splitting under simulated solar light. Appl. Catal. B Environ. 304, 121000. 10.1016/j.apcatb.2021.121000

[B30] Xiaoquan L. Zhang H. Liu X. Yuan H. Yang J. (2007). Investigation of the antioxidant property of ascorbic acid. J. phys. chemis. 111 (41), 14998–15002. 10.1021/jp072551i

[B31] Zhao W.-W. Xu J.-J. Chen H.-Y. (2015). Photoelectrochemical bioanalysis: the state of the art. Chem. Soc. Rev. 44 (3), 729–741. 10.1039/c4cs00228h 25223761

[B32] Zhou C.-M. Zhao S.-H. (2024). Evaluation of the value of combined detection of tumor markers CA724, carcinoembryonic antigen, CA242, and CA19-9 in gastric cancer. World J. Gastrointest. Oncol. 16 (5), 1737–1744. 10.4251/wjgo.v16.i5.1737 38764828 PMC11099441

[B33] Zhou M. Pu Y. Wu Q. Wang P. Liu T. Zhang M. (2020). 2D hexagonal SnS2 nanoplates as novel co-reaction accelerator for construction of ultrasensitive g-C3N4-based electrochemiluminescent biosensor. Sensors Actuators B Chem. 319, 128298. 10.1016/j.snb.2020.128298

