# Supplementary material for: Ultrasensitive photoelectrochemical immunosensor based on floral cluster SnS2/ZnCdS heterostructure for the detection of CA199
Source: Front Bioeng Biotechnol. 2025 Jun 11;13:1584456. doi: 10.3389/fbioe.2025.1584456 (PMC12187654; doi:10.3389/fbioe.2025.1584456)
Supplement: Supplementary file 1 [file DataSheet1.docx]

Supplementary Material

A photoelectrochemical immunosensor based on Floral cluster SnS_2_/ZnCdS heterostructure for the detection of CA199

Hui Zhou^1^, Qingqing Guo^2^, Xin Zhang^1^, Tingting Chu^1^, Wen Zhang^1^, Qing Liu^3^, Linlin Cao^1^*

*^1^ Zibo Central Hospital Affiliated to Binzhou Medical University, Zibo, Shandong, China.*

*^2^ PKUCare Luzhong Hospital, Zibo, Shandong, China.*

*^3^ School of Chemistry and Chemical Engineering, Shandong University of Technology, Zibo, Shandong, China.*

*** Correspondence:**Linlin Cao
[caolinlin0101@163.com](mailto:caolinlin0101@163.com)

# Supplementary Tables

**Table S1** The *R*_et_ of EIS.

| Working electrode | *R*_et_ (Ω) | *R*_s_ (Ω) | *C*_dl_ (Ω) | *Z*_w_ (Ω) |
| --- | --- | --- | --- | --- |
| (a) ITO | 0 | - | - | - |
| (b) ITO/SnS_2_/ZnCdS | 31.6 | 49.1 | 8.49×10^-6^ | 1956 |
| (c) ITO/SnS_2_ | 34.0 | 48.6 | 1.18×10^-5^ | 1241 |
| (d) ITO/ZnCdS | 38.2 | 55.0 | 9.04×10^-6^ | 2112 |

**Table S2** The *R*_et_ of EIS.

| Working electrode | *R*_et_ (Ω) | *R*_s_ (Ω) | *C*_dl_ (Ω) | *Z*_w_ (Ω) |
| --- | --- | --- | --- | --- |
| (a) ITO | 0 | - | - | - |
| (b) ITO/SnS_2_/ZnCdS | 31.6 | 49.1 | 8.49×10^-6^ | 1956 |
| (c) ITO/SnS_2_/ZnCdS/TGA | 44.6 | 61.0 | 1.22×10^-5^ | 1989 |
| (d) ITO/SnS_2_/ZnCdS/TGA/EDC/NHS | 54.1 | 48.0 | 7.05×10^-6^ | 2687 |
| (e) ITO/SnS_2_/ZnCdS/TGA/EDC/NHS/anti-CA199 | 86.4 | 60.9 | 8.75×10^-6^ | 2091 |
| (f) ITO/SnS_2_/ZnCdS/TGA/EDC/NHS/anti-CA199/BSA | 152.5 | 63.3 | 6.89×10^-6^ | 3889 |
| (g) ITO/SnS_2_/ZnCdS/TGA/EDC/NHS/anti-  CA199/BSA/CA199 | 174.5 | 51.7 | 9.38×10^-6^ | 2645 |

# Supplementary Figures

**Figure S1 UV-Vis-DRS of ZnCdS.**

**
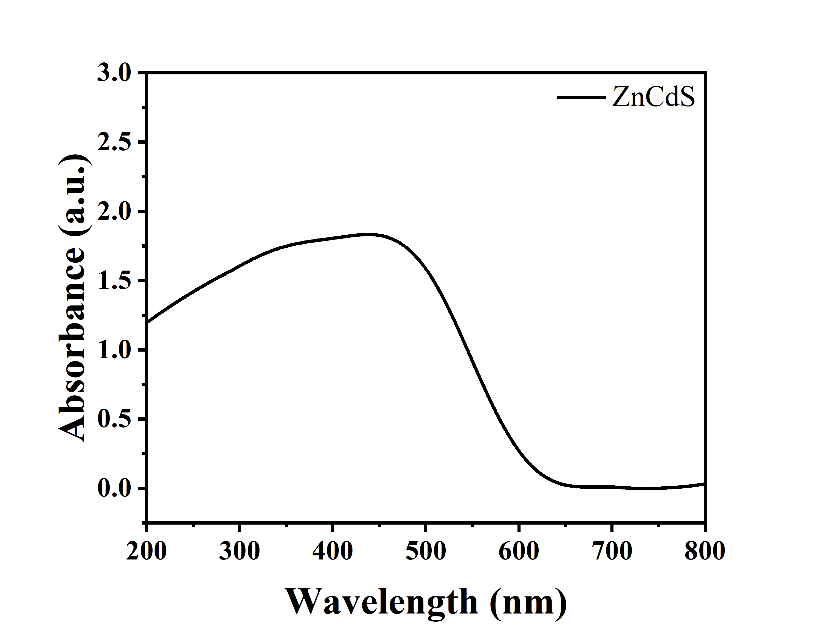
**

**Figure S2 (A)** **The band gap diagram of SnS₂; (B) The band gap diagram of ZnCdS; (C)** **Mott-Schottky curve of SnS_2_; (D) Mott-Schottky curve of ZnCdS.**


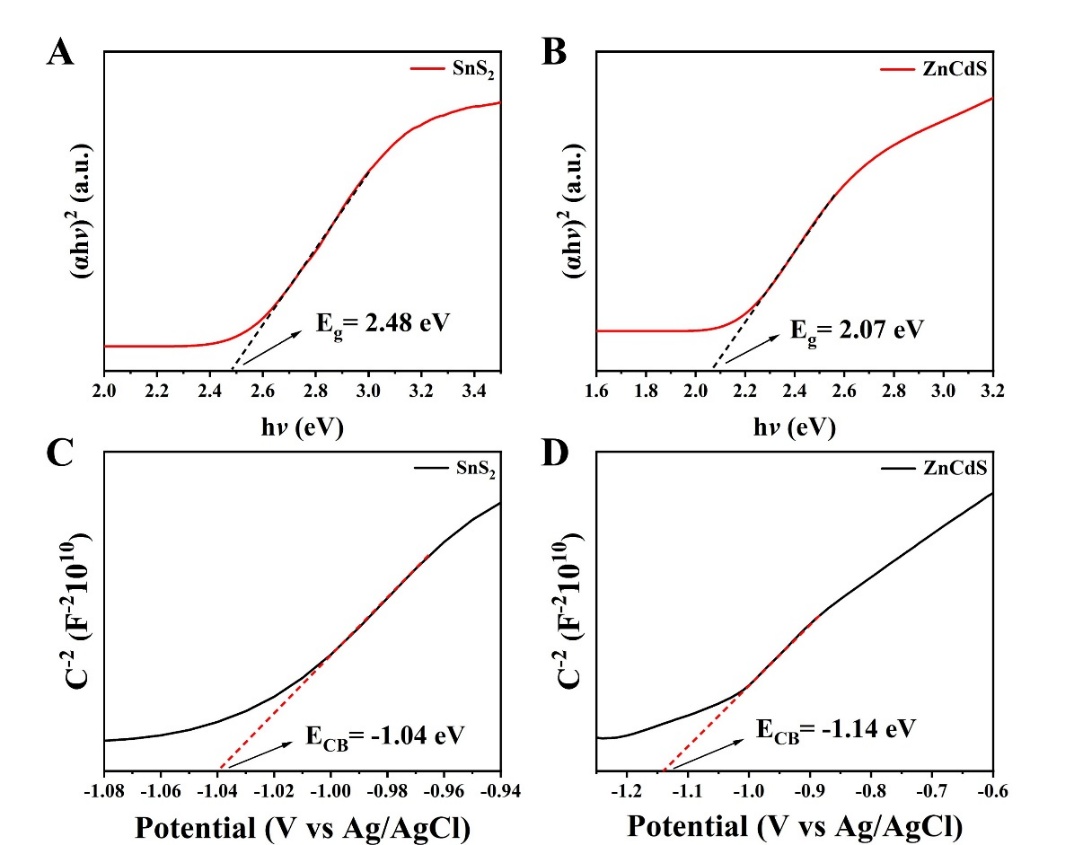


# The calculation of LOD:

The International Union of Pure and Applied Chemistry (IUPAC) stipulates that the LOD was taken as the lowest concentration of an analyte in a sample that could be detected. The *x_L_* was derived from the smallest measure that could be detected with reasonable certainty for a given analytical procedure. The value of *x_L_* was given by the equation *x_L_* = *x_b1_* + *ks_b1_*, where *x_b1_* was the mean of the blank measures, *s_b1_* was the standard deviation of the blank measures and *k* was a numerical factor chosen according to the confidence level desired. A value of 3 for k in the equation was strongly recommended, and it corresponded to a confidence level of about 90 % in a practical sense. The calibration curve obtained by the immunosensor was ΔI (μA) = 2.1996 – 0.4805 × lg c (U/mL). Hence, the *c_L_* was 1.00 × 10^-3^ U/mL by the formula *c_L_* = 10^(^*^xL^* ^- 2.1996) / (-0.4805)^.
